# The Effect of Light Emitting Diodes (LEDs) Spectra on Growth, Morphological and Genetic Stability of *Eurycoma longifolia* Hairy Root Cultures through ISSR and DAMD Analysis

**DOI:** 10.21315/tlsr2025.36.2.9

**Published:** 2025-07-31

**Authors:** Mahmoud Ali Khalaf Abushattal, Sani Sale, Sreeramanan Subramaniam, Nor Hasnida Hassan, Mohamad Fadhli Mad’ Atari

**Affiliations:** 1 School of Biological Sciences, Universiti Sains Malaysia, 11800 USM, Pulau Pinang, Malaysia; 2 Department of Botany, Gombe State University, Gombe P.M.B 127, Nigeria; 3 Centre for Chemical Biology, Universiti Sains Malaysia, 11900 Bayan Lepas, Pulau Pinang, Malaysia; 4 Department of Biology, Faculty of Science and Technology, Universitas Airlangga, Surabaya 60115, Indonesia; 5 Forest Biotechnology Division, Forest Research Institute Malaysia, 52109 Kepong, Selangor, Malaysia

**Keywords:** Tongkat Ali, Hairy Root Culture, Medicinal Plant, Genetic Stability, Tongkat Ali, Kultur Akar Rerambut, Tumbuhan Ubatan, Kestabilan Genetik

## Abstract

Light quality significantly influences plant growth and development by interacting with photoreceptors, leading to reversible and irreversible outcomes. This study provides novel insights into the species-specific effects of light-emitting diodes (LEDs) on the morphological characteristics and genetic stability of *Eurycoma longifolia* hairy root cultures (ELHRCs) under different light qualities. LED treatments included white (400 nm–700 nm), blue (440 nm), mint green (530 nm), red (660 nm) and a combination of blue with red (1:1) (440 nm + 660 nm) applied for 8, 10 and 12 weeks. Morphological changes and growth were assessed alongside genetic stability through direct amplification of minisatellite DNA regions (DAMD) and inter-simple sequence repeat (ISSR) markers analysis using 12 primers. The results showed genetic similarity of 90.6% after 8 weeks and 100% after 10 and 12 weeks (DAMD) and 100%, 98.2% and 90.3% after 8, 10 and 12 weeks, respectively (ISSR) under all LED treatments, confirming the genetic stability of the hairy roots. Additionally, the study demonstrated how spectral quality influences hairy roots growth and morphology. The high percentage of genetic similarity highlights LEDs as a promising tool for *in vitro* culture of ELHRCs. These findings represent the first comprehensive report on the combined effects of LED spectral quality on growth, morphological changes and genetic stability in ELHRCs.

## Abstrak

Kualiti cahaya secara signifikan mempengaruhi pertumbuhan dan perkembangan tumbuhan melalui interaksi dengan fotoreseptor, yang menghasilkan kesan boleh balik dan tidak boleh balik. Kajian ini memberikan perspektif baharu mengenai kesan spesifik spesies diod pemancar cahaya (LED) terhadap ciri morfologi dan kestabilan genetik kultur akar berbulu *Eurycoma longifolia* (ELHRCs) di bawah pelbagai kualiti cahaya. Rawatan LED yang digunakan termasuk putih (400 nm–700 nm), biru (440 nm), hijau pudina (530 nm), merah (660 nm) dan gabungan biru dan merah (1:1) (440 nm + 660 nm) yang diaplikasikan selama 8, 10 dan 12 minggu. Perubahan morfologi dan pertumbuhan dinilai bersama kestabilan genetik menggunakan analisis penanda penguatan langsung kawasan DNA minisatelit (DAMD) dan jujukan antara mudah (ISSR) dengan 12 primer. Keputusan menunjukkan persamaan genetik sebanyak 90.6% selepas 8 minggu dan 100% selepas 10 dan 12 minggu (DAMD), serta 100%, 98.2% dan 90.3% masing-masing selepas 8, 10 dan 12 minggu (ISSR) di bawah semua rawatan LED, mengesahkan kestabilan genetik akar berbulu. Selain itu, kajian ini menunjukkan bagaimana kualiti spektrum LED mempengaruhi pertumbuhan dan morfologi akar berbulu. Peratusan persamaan genetik yang tinggi menonjolkan LED sebagai alat yang berpotensi untuk kultur *in vitro* ELHRCs. Penemuan ini merupakan laporan komprehensif pertama mengenai kesan gabungan kualiti spektrum LED terhadap pertumbuhan, perubahan morfologi dan kestabilan genetik dalam ELHRCs.

**Highlights**

- Mint green LED (530 nm) promotes biomass accumulation: Among all tested light treatments, mint green LED significantly enhanced fresh and dry weight accumulation in Eurycoma longifolia hairy root cultures after 10 weeks of culture.
- LED treatments maintain genetic stability: Genetic stability analysis using DAMD and ISSR markers confirmed high genetic similarity (> 90%) across all LED treatments, indicating that LED exposure does not induce somaclonal variation in E. *longifolia* hairy root cultures.
- Spectral quality influences root morphology and development: Different LED wavelengths affected root growth patterns and overall morphology, demonstrating specific responses to spectral quality indicating a possible stress response or metabolic shift.

## INTRODUCTION

Light quality significantly influences morphogenesis, growth and differentiation in plant cells, tissues, and organ cultures (Araújo *et al.* 2021; Nery *et al.* 2021). Light-emitting diodes (LEDs) are increasingly used in *in vitro* tissue culture due to their ability to provide tailored light spectra, optimising plant growth and metabolic efficiency (Jung *et al.* 2021; Zieliñska *et al*. 2020). Unlike traditional light sources such as halogen lamps and fluorescent tubes, LEDs are mercury-free, energy-efficient and environmentally sustainable, making them ideal for plant breeding applications (Bachouch *et al.* 2021).

LEDs are excellent artificial lighting due to their short wavelength and their ability to provide tailored coloured light that is efficiently absorbed by the plant photoreceptors (Batista *et al.* 2018; Chaves *et al.* 2020; Paradiso & Proietti 2022). The LED light prevents overheating of the developing material, saving energy and the environment (Doulos *et al.* 2020). LEDs also have simpler driver electronics, longer lifetime, reliability and lower maintenance costs (Niangoran *et al.* 2016; Bachouch *et al.* 2021).

The physiological and morphological responses of plants to different light wavelengths depend on the selective activation of photoreceptors (Coelho *et al*. 2021). For instance, blue light enhances root biomass in *Astragalus membranaceous* (Gai *et al.* 2022), while green light promotes root regeneration (Li *et al.* 2021). Similarly, the combination of specific wavelengths has been shown to influence hairy root morphology in *Salvia miltiorrhiza* (Chen *et al.* 2018). These findings underscore the importance of spectral composition in promoting optimal growth and root development in tissue culture systems.

However, somaclonal variation, which occurs during *in vitro* culture, can compromise clonal fidelity and genetic stability. Factors such as plant growth regulators, subcultures and light conditions can influence genetic integrity (Boldaji *et al.* 2021). Intense or fluctuating lighting can induce oxidative stress, affecting DNA replication and cell division (Krishna *et al.* 2016; Bidabadi & Jain 2020). Molecular markers such as inter-simple sequence repeats (ISSR) and direct amplification of minisatellite DNA (DAMD) have been widely used to assess genetic variation and stability in tissue culture systems (Nisa *et al.* 2019; Orłowska 2021).

*Eurycoma longifolia* (Tongkat Ali), a medicinal herb widely used in Southeast Asia, is valued for its pharmacological properties, including antioxidant, anti-inflammatory, anticancer and immunomodulatory effects (Rehman *et al.* 2016; Yunos *et al.* 2022; Sale *et al.* 2023). Hairy root culture of *E. longifolia* offers a sustainable approach to producing its bioactive compounds (Nazirah *et al.* 2018). Given the importance of light quality for plant growth, this study for the first time aimed to evaluate the effects of different LED wavelengths white (400 nm–700 nm), blue (440 nm), mint green (530 nm), red (660 nm) and a combination of blue with red (1:1) on growth, morphology and genetic stability of *E. longifolia* hairy root cultures (ELHRCs).

## MATERIAL AND METHODS

### Plant Material and Growth Conditions

**Figure 1:**
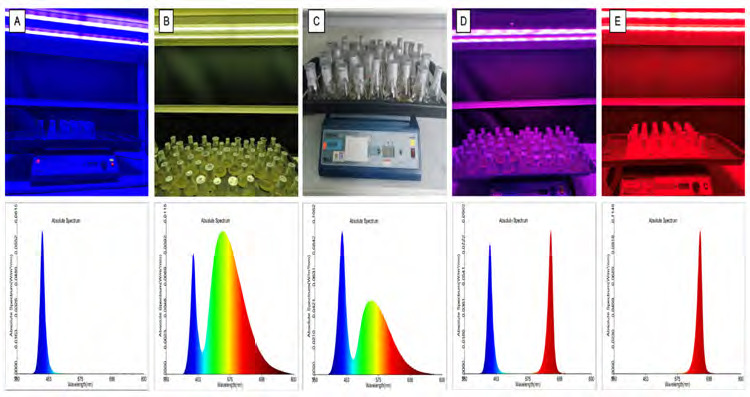
ELHRCs growing in various LED Treatments light spectra of five types of LED used in the experiment: (A) blue, (B) mint green, (C) white, (D) blue plus red (1:1) and (E) red.

The ELHRCs were obtained from the Forest Research Institute Malaysia and maintained at the School of Biological Sciences, Universiti Sains Malaysia were used for this study. Murashige and Skoog (MS) basal medium with a pH of 5.8 was used as the culture medium and autoclaved at 121°C for 15 min. The cultures were incubated at 22 ± 2°C in the dark on an orbital shaker (110 rpm) with 16/8 h light/dark photoperiod. The morphology, growth and genetic stability of ELHRCs were analysed using different light spectra, including white (400 nm–700 nm) [W], blue (440 nm) [B], red (660 nm) [R], blue plus red (1:1) (440 nm + 660 nm) [BR] and mint green [MG] (530 nm). The photosynthetic photon flux densities (PPFD) of these LED lights were determined as follows: W (44.38 μmol m^−2^s^−1^), R (14.24 μmol m^−2^s^−1^), B (6.40 μmol m^−2^s^−1^), BR (17.63 μmol m^−2^s^−1^) and MG (7.90 μmol m^−2^s^−1^). The control was represented white light. Hairy root samples were collected after 8, 10 and 12 weeks from each treatment. The analyses were conducted for genetic similarity using directed amplification of DNA from minisatellite regions (DAMD) and genetic inter simple sequence repeats (ISSR) markers (Fig. 1).

### Measurement of Biomass and Examination of Growth Morphology

To measure the biomass, three bottles of ELHRCs were harvested from each treatment at 8 weeks, 10 weeks and 12 weeks after culture. Fresh weight was taken directly, and the samples were air-dried at 37°C until constant weights were attained for dried biomass. The growth and morphology, including hairy root colour and texture, were examined physically and documented with the help of a camera.

### DNA Extraction Procedure

DNA extraction was performed according to the instructions of the NucleoSpin® Plant II Genomic DNA Purification Kit. Crush 100 mg of fresh root samples in a cold mortar and pestle and then add 300 μL PL2 nuclear lysis solution and 10 μL RNase solution to homogenise. The mixture was placed in a sterile microcentrifuge tube and incubated at 65°C for 10 min after a short vortex. Then add 75 μL PL3, mix and incubate for 5 min on ice. To filter the crude lysate, centrifuge at 11,000 ×g for 2 min. The DNA-containing supernatant was transferred to a new microcentrifuge tube containing 450 μL BC. The supernatant was discarded after centrifugation at 11,000 ×g for 1 min. The DNA pellet was washed three times: first with 400 μL PW1, centrifuged at 11,000 ×g for 1 min, then with 700 μL PW2 and finally with 200 μL PW2 for 2 min. To elute the DNA, add 50 μL of buffer PE (65°C) and centrifuge at 11,000 ×g for 1 min, repeating this step twice. The isolated DNA was stored at −40°C.

### PCR Amplification of Genomic DNA Using DAMD – DNA Method

To determine the genetic stability of ELHRC, 12 DAMD primers were evaluated (Table 1). Devi *et al.* (2014) performed DNA amplification using the first base Malaysia DAMD primers. PCR amplification was performed in a 200 μL tube (Axygen Inc., California, USA) with a 25 μL reaction mixture, 12.5 μL GoTaq® Green Master Mix, 2.5 μL primers, 2 μL template DNA and 8 μL nuclease-free water. The PCR process was performed using the MyCycler™ Thermal Cycler (Bio-Rad Laboratories, Inc., USA). The PCR amplification conditions were denaturation at 94°C for 2 min, 40 cycles at 92°C for 1 min, annealing at Tm – 5°C for 2 min, 72°C and a final extension cycle at 72°C for 10 min.

### PCR Amplification of Genomic DNA Using ISSR – DNA Method

In the present study, the genetic stability of ELHRCs was analysed using 12 ISSR primers (Devi *et al.* 2014) (Table 2). PCR amplification was performed in a 200 μL tube (Axygen Inc., California, USA) with 12.5 μL GoTaq® Green Master Mix, 2.5 μL primers, 2 μL template DNA and 8 μL nuclease-free water. PCR was performed using the MyCycler™ Thermal Cycler (Bio-Rad Laboratories Inc., USA). PCR amplification conditions started with 3 min of denaturation at 94°C. Each cycle included 1 min of denaturation at 94°C, 1 min of annealing (estimated at Tm–5°C), 2 min of extension at 72°C and 10 min of extension at 72°C, which was repeated 40 times.

### Gel Electrophoresis Analysis

Gel electrophoresis separated the PCR products. The agarose (1.5%) was heated in 40 mL of 1×Tris-Borate-EDTA (TBE) buffer in the microwave. It was cooled to room temperature and stained with 2 μL of Red-safe nucleic acid staining solution (iNtRON Biotechnology, Mini Gel Caster, Bio-Rad Laboratories, USA). The mixture solidified at room temperature for 20 min. The gel was solidified using the Wide Mini Sub-Cell® GT Agarose Gel Electrophoresis System from Bio Rad Laboratories, Inc. in the USA, with 1× TBE buffer. The wells contained Thermo Scientific Gene Ruler 1kb (Lithuania) and 100 bp Plus. Add 6 μL of the PCR products to each well. After connecting the PowerPac™ Basic Power Supply (Bio Rad Laboratories Inc., USA), the electrophoresis system ran at 70 V for 75 min. The UVIdoc HD5 Gel Imaging System was used to observe the different DNA bands after exposure to ultraviolet (UV) light.

### Determination of Polymorphism Analysis

The migration pattern of amplified PCR products from hormone-free MS medium *in vitro* ELHRCs treated with different LEDs was evaluated by comparing the presence or absence of bands with the separation pattern of *in vitro* ELHRCs cultured in the white. The identification of distinct and reproducible bands was done manually, assigning a score of 0 for the absence of bands and a score of 1 for the presence of bands. The similarity index was calculated using a specific method developed by Harirah and Khalid (2006):


$$\text{Similarity Index }(\text{SI})=\frac{2\text{Nxy}}{\text{Nx}+\text{Ny}}$$

where, Nxy = number of monomorphic bands between the control and treatment groups; Nx = total number of bands in the control group and Ny = total number of bands in the treatment group.

### Statistical Analysis

All experiments were performed in three biological replicates. The data from all experiments conducted were analysed using (one-way) analysis of variance (ANOVA) followed by Duncan’s post hoc test at *p* ≤ 0.05 using the Statistical Package for the Social Sciences (SPSS) version 28.0. The results of fresh and dry biomass were expressed as mean values and their standard errors (SE) using MS Excel software.

## RESULTS AND DISCUSSION

### Effects of LED Treatments on the Growth and Morphology of ELHRCs

**Figure 2:**
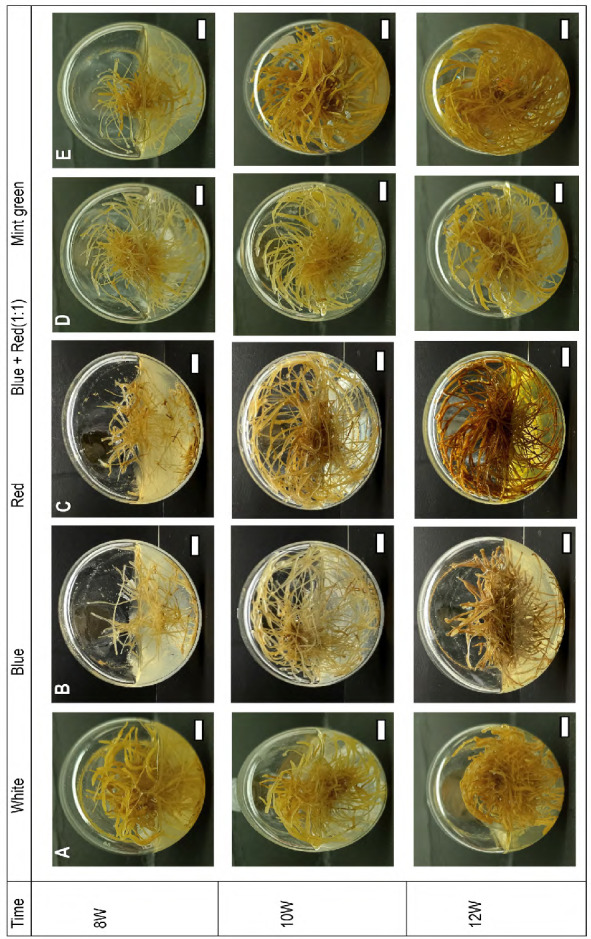
Morphology of *E. longifolia* hairy root cultures in different LEDs: at 8 weeks, 10 weeks and 12 weeks’ interval (scale bar = 1.0 cm)

*E. longifolia* hairy root cultures (ELHRCs) are exposed to LED lamps with different spectral for 8 weeks, 10 weeks and 12 weeks. The different LED treatments, namely red (R), blue (B), mint green (MG), blue plus red 1:1 (BR) and white (W) as a control, had a significant effect on the growth and morphological characteristics of ELHRCs, as shown in Fig. 2 and Table 3.

The colour of ELHRCs varied depending on the LED treatments and culture duration (Fig. 2). After 8 weeks of treatment, the hairy roots displayed a yellow colour in B light. Furthermore, under W, R, BR and MG light treatments, the hairy roots appear yellowish-orange in colour. After 10 weeks, the hairy roots appear yellow under W, B and BR light treatments and yellowish-orange in colour under MG light treatment. After 12 weeks, the hairy roots colour under R-light was orange, whereas W, B and BR lights the roots were yellow, and MG light the roots appeared yellowish-orange. The ELHRCs has a dark brown colour that is more obvious at 12 weeks in R light treatment. Therefore, the duration of light treatments affects the colour of *E*. *longifolia* hairy roots.

The results in Table 2 show that after 8 weeks of the experiment, the highest dry weight was recorded under W light, while the dry weight of hairy roots obtained under R, B, BR and MG lights was significantly lower, while no significant difference was observed in term of fresh weight (FW). After 10 weeks of culture, R light resulted significantly less FW compared to MG light followed by W, B and BR lights. The use of light of different wavelengths modified the dry weight (DW) of *E*. *longifolia* roots. On the other hand, no significant difference was observed in FW and DW after 12 weeks of treatment.

Despite this, FW accumulation was consistently higher under W light after 8 and 12 weeks. However, after 10 weeks, FW under MG light was 1.2 times higher than under W light, and DW was 1.08 times higher under MG light compared to W light. These results, effect of LED treatments on the ELHRCs of fresh and dry weight as shown in Table 3, indicate that MG light is optimal for biomass production in ELHRCs after 10 weeks of culture.

These findings align with previous studies. For example, Nazirah *et al.* (2018) reported higher biomass production in *E. longifolia* hairy root cultures grown in the dark for 10 weeks. In addition, studies on other species, such as ginseng (Yu *et al.* 2005) and beetroot (Shin *et al.* 2003), found that red and far-red light produced the highest biomass. Interestingly, in ELHRCs, R light resulted in significantly lower biomass than MG light after 10 weeks.

However, blue light has also been reported to enhance hairy root biomass in other species. Gai *et al*. (2022) observed that blue light produced 1.4 times more biomass in *A. membranaceus* hairy roots compared to dark after 55 days. Similarly, Jiao *et al*. (2023) reported a 1.86-fold increase in biomass under blue light in *Isatis tinctoria* hairy roots cultured for 50 days. Additionally, Zhang *et al*. (2020) found that the combination of blue and red light significantly influenced the morphology and growth of *S. miltiorrhiza* hairy roots. However, Chen *et al.* (2018), the duration of light treatment can influence the colour of *S. miltiorrhiza* hairy roots. Similarly, Mukherjee *et al.* (2016) observed that *Daucus carota* hairy roots turned green under continuous illumination. The results of this study show that MG monochromatic LEDs produce higher biomass after 10 weeks of culture. This could be due to the mixture’s synergistic effects, which consist of light essential for most plant physiological functions. In addition, the ELHRCs after 12 weeks show no significant difference between LED treatments, might be because of the consumption of nutrients in the medium.

### DAMD–DNA Analysis

**Figure 3:**
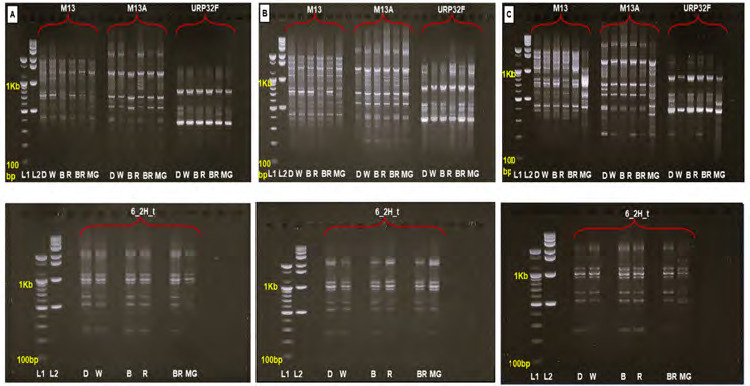
Influence of different LED treatments on DAMD profiles of *in vitro Eurycoma longifolia* hairy root cultures (ELHRCs). DAMD primers (URP32F, M13, M13A, and 6_2H_t) were used. (A) ELHRCs at 8 weeks, (B) ELHRCs at 10 weeks and (C) ELHRCs at 12 weeks of treatment. *Abbreviations* : (L1) 100 bp ladder, (L2) 1kb ladder, (D) Dark, (W) White, (B) Blue, (R) Red, (BR) Blue plus Red (1:1) and (MG) Mint Green.

A total of 12 DAMD primers were initially screened to analyse the genomic DNA extracted from ELHRCs samples. Of these, four primers M13, M13A, URP32F and 6_2H_t produced well-defined and reproducible banding patterns across all treatments (R, B, BR, MG and control W). The amplified DNA fragments ranged in size from 200 bp to 2,900 bp (Fig. 3A–C; Tables 4–6).

The DAMD assay aimed to examine DNA band variation, reveal the degree of polymorphism, and assess genetic stability of ELHRCs grown under different LED treatments (Fig. 1). Monomorphic bands were consistent with the control, while polymorphic bands showed deviations. Under all LED treatments, ELHRCs demonstrated high genetic stability, producing monomorphic bands with the selected primers (Fig. 3A–C).

Primer M13A generated the highest number of amplified DNA bands (12) after 8 weeks under R light, with band sizes ranging from 300 bp to 2,500 bp. At this stage, primers M13 and 6_2H_t achieved a similarity index (SI) of 1.0 in all LED treatments (Table 4). After 10 weeks, primer M13 produced the most amplified bands (14), ranging from 350 bp to 2000 bp, with SI 1.0 observed in W, B and BR treatments. Similarly, primers URP32F and 6_2H_t-maintained SI 1.0 in all treatments (Table 5). By 12 weeks, M13 produced the highest number of bands (13) under B light, while primer URP32F continued to exhibit SI 1.0 across all treatments (Table 6).

Particularly, variations in SI were observed with some primers under specific conditions. After 8 weeks, primer M13A yielded SI values of 0.89 and 0.95 for B and MG light treatments, respectively, due to the deletion of 4 and 3 bands. Primer URP32F showed SI values of 0.89, 0.91 and 0.91 for B, BR and MG light treatments, respectively, caused by one band deletions (Table 4). After 10 weeks, primer M13 recorded SI values of 0.96 for R and MG light treatments due to single-band deletions, while M13A produced SI values of 0.96 for R, B and MG, reflecting single-band additions. BR treatment showed an SI of 0.92 due to the deletion of two bands (Table 5). At 12 weeks, primer M13 had SI values of 0.96 and 0.92 for B and MG light treatments due to one added band and two deleted bands, respectively. Primer M13A recorded an SI of 0.95 for BR light treatment, reflecting the deletion of one band (Table 6).

### ISSR-DNA Analysis

**Figure 4:**
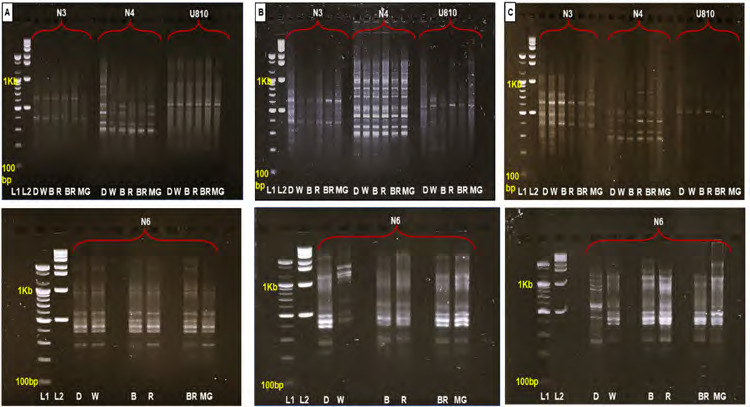
Influence of different LED treatments on ISSR profiles of *in vitro Eurycoma longifolia* hairy root cultures (ELHRCs). ISSR primers (N3, N4, U810, and N6) were used. (A) ELHRCs at 8 weeks, (B) ELHRCs at 10 weeks and (C) ELHRCs at 12 weeks of treatment. Abbreviations: (L1) 100 bp ladder, (L2) 1kb ladder, (D) Dark, (W) White, (B) Blue, (R) Red, (BR) Blue plus Red (1:1) and (MG) Mint Green.

For ISSR analysis, 4 primers N3, N4, U810 and N6 were selected from 12 primers based on their ability to generate clear and reproducible banding patterns under all treatments (R, B, BR, MG and W). The amplified DNA fragments ranged in size from 200 bp to 2,900 bp (Fig. 4(A–C); Tables 7–9).

ISSR analysis further evaluated DNA band variation, focusing on the degree of polymorphism and genetic stability of ELHRCs under different LED treatments (Fig. 1). Monomorphic bands were consistent with the control lane, while polymorphic bands deviated. High genetic stability was observed across all treatments, with monomorphic bands produced by the 4 primers (Fig. 4A–C).

Primer N6 generated the highest number of amplified bands (10) after 8 weeks, ranging from 300 bp to 2,900 bp, with an SI of 1.0. Other primers (N3, N4 and U810) also achieved SI 1.0 in all treatments (Table 7). After 10 weeks, primer N4 produced the highest number of bands (13), ranging from 350 bp to 2,000 bp, while primers N3 and N6 maintained SI 1.0 across all LED treatments. Primer N4 achieved SI 1.0 in R and B light treatments (Table 8). After 12 weeks, primer N6 again produced the highest number of bands (13), while primer U810 maintained SI 1.0 across all LED treatments (Table 9).

However, variations in SI values were observed under specific conditions. After 8 weeks, primer N4 showed SI values of 0.95 for B and MG treatments, reflecting the deletion of one band (Table 7). At 10 weeks, primer U810 displayed SI values of 0.33 for R and BR light treatments and 0.89 for MG light, resulting from the deletion of four, four and one bands, respectively. Primer N4 recorded SI values of 0.92 and 0.96 for BR and MG light treatments, respectively, due to band deletions (Table 8). After 12 weeks, primer N3 showed SI values of 0.95, 0.84 and 0.84 for R, BR and MG light treatments, reflecting the deletion of one, three and three bands, respectively. Primer N4 recorded SI values of 0.77, 0.83 and 0.91 for R, BR and MG light treatments, resulting from deletions of three, two and one bands, respectively. Primer N6 achieved SI values of 0.92 for R and BR light treatments due to the deletion of two bands each (Table 9).

Genetic stability or variation in response to environmental factors can be effectively assessed using molecular markers such as RAPD, ISSR, DAMD, SSR and minisatellite DNA. SSR and microsatellite markers have been isolated and characterised in natural populations of *E. longifolia* (Tnah *et al*. 2011; Lee *et al*. 2018), while RAPD markers have been used to characterise and compare these populations (Razi *et al.* 2013). These foundational studies support the application of molecular markers like ISSR and DAMD in assessing the genetic stability of *in vitro E. longifolia* hairy root cultures (ELHRCs). In this study, ISSR and DAMD markers were employed to evaluate genetic stability in ELHRCs subjected to different LED treatments after 8, 10 and 12 weeks.

Various studies have demonstrated the effectiveness of ISSR, DAMD and RAPD markers in detecting genetic variation. For instance, Lamare and Rao (2015) evaluated genetic diversity in *Musa acuminata* cultivars using these markers, reporting an overall polymorphism of 90.06%. In contrast, the ELHRCs in our study exhibited significantly lower polymorphism levels: 17.2%, 7.5% and 10% for DAMD primers, and 7.9%, 19.6% and 14.5% for ISSR primers after 8, 10 and 12 weeks, respectively. According to Zoghlami *et al*. (2012), genotypes with more than 90% genetic similarity are considered genetically stable. Our results indicate that LED-treated ELHRCs maintained genetic stability, with SI exceeding 90% across all treatments (Tables 4–9).

Tissue-engineered plants are susceptible to molecular changes due to various factors, including plant growth regulators (PGRs), chemicals in the culture medium and light spectra. These changes may lead to genetic mutations, impacting plant chemistry, structure and cellular composition (Long *et al.* 2022). For ELHRCs, such morphological variations could potentially result from epigenetic modifications. Therefore, molecular testing to confirm genetic stability is essential to develop advanced methods for detecting somaclonal variations. Identifying and managing somaclonal variations are critical for ensuring the genetic stability of micropropagated plants (Leva & Petruccelli 2012).

Plant morphogenesis, including processes such as root elongation, leaf expansion and metabolic changes, depends on the availability and quality of light (Paradiso & Proietti 2022). Studies by Olle and Viršile (2013), Mills and Dunn (2016) and Monostori *et al*. (2018) highlight the influence of photosynthetically active radiation (PAR) on plant growth under controlled conditions. LEDs have emerged as an efficient light source, enhancing plant cell properties (Gnasekaran *et al.* 2022). Park *et al.* (2010) suggested that LEDs minimise somaclonal variations during mass propagation, offering growth characteristics comparable to fluorescent lights. Thus, LEDs constructed from visible light wavelengths are a safe and economical option for culturing ELHRCs.

Specific wavelengths, such as red and blue light, are known to improve plant productivity (Olle & Viršile 2013; Borowski *et al.* 2015; Park & Runkle 2018). For instance, light directly influences gene expression at various developmental stages, as shown by Torres et al. (2019). Such genetic-level changes may explain variations in photosynthetic parameters and plant yields (Cioć *et al.* 2018). In this study, ELHRCs exposed to MG light exhibited higher biomass yields, while all LED treatments influenced distinct morphological traits. Gene expression or epigenetic analyses could further elucidate the mechanisms underlying these changes.

The reliability of molecular analyses also plays a role in assessing genetic stability. Parab *et al.* (2021) suggested that error dynamics during PCR amplification can reduce variability in SI values. For ELHRCs, the low percentages of SI were observed for the U810 primer in R and BR after 10 weeks, possibly due to the same explanation as in Table 7. However, genetic stability was high, 90.6% after 8 weeks and 100% after 10 and 12 weeks for DAMD primers, and 100%, 98.2% and 90.3% after 8 weeks, 10 weeks and 12 weeks, respectively, for ISSR primers as shown in Table 10.

These findings confirm the genetic stability of ELHRCs during LED treatment and align with studies on other plant species. For example, Purayil *et al.* (2018) analysed date palm cultivars using ISSR and DAMD markers, reporting 85.45% polymorphism. Similarly, genetic differences in *Douglas fir*, *Sugi* and *melon* have been analysed using ISSR and RAPD markers, showing high variation (Daryono *et al.* 2019). Conversely, studies on *Platanus acerifolia* reported genetic stability in micropropagated plants (Huang *et al.* 2009; Matsumoto *et al.* 2013). Overall, the results demonstrate that potential of LED lights as a viable and efficient tool for enhancing plant growth and maintaining genetic stability in *in vitro* ELHRCs.

## CONCLUSION

The results of this study showed that light quality, defined by specific wavelengths emitted by LEDs, has a significant effect on the growth, morphological characteristics, and maintenance of the genetic stability of *Eurycoma longifolia* hairy root cultures (ELHRCs). After 10 weeks of culture, hairy roots under MG light produce the highest fresh and dry biomass. This was supported by a high genetic similarity between the LED-treated cultures as evidenced by DAMD and ISSR markers analyses after 8, 10, and 12 weeks of treatment. In addition, ELHRCs under all LED treatments had an average genetic similarity index above 90%, indicating genetic stability.

## Figures and Tables

**Table 1:** List of DAMD primers.

Primer	Genomic sequence (3′ 5′)	G + C content (%)	Tm (°C)	Annealing temp.
URP30F	GGA CAA GAA GAG GAT GTG GA	50.00	53.9	48.9
URP32F	TAC ACG TCT CGA TCT ACA GG	50.00	53.0	48.0
URP38F	AAG AGG CAT TCT ACC ACC AC	50.00	54.5	49.5
HBV5	GGT GTA GAG AGG GGT	60.00	49.0	45.0
HVR	CCT CCT CCC TCC T	69.23	47.6	42.6
HBV3	GGT GAA GCA CAG GTG	60.00	50.00	45.0
6.2H(+)	AGG AGG AGG GGA AGG	66.67	52.4	47.4
M13	GAG GGT GGC GGC TCT	73.33	57.9	52.9
M13A	GAG GGT GGC GGT TCC T	68.75	57.6	52.6
HVA	AGG ATG GAA AGG AGG C	56.25	51.0	46.0
HVV	GGT GTA GAG AGG GGT	60.00	49.0	44.0
6_2H_t	AGG AGG AGG GGA AGG	66.67	52.4	47.4

**Table 2:** List of ISSR primers.

Primer	Genomic sequence (3′ 5′)	G + C content (%)	Tm (°C)	Annealing temp.
N1	ACA CAC ACA CAC ACA CT	47.06	51.4	49.4
N2	TGT GTTG TGT GTG TGT GA	44.44	51.3	49.3
N3	GAG AGA GAG AGA GAG AYT	44.44	47.4	42.4
N4	CAC ACA CAC ACA GG	57.14	46.2	41.2
N5	CAC ACA CAC ACA AC	50.00	43.6	38.6
N6	CAC CAC CAC GC	72.73	44.7	39.7
N8	CAC ACA CAC ACA GT	50.00	44.7	39.7
N9	ACA CAC ACA CAC ACA CAG	50.00	52.9	47.9
N10	ACA CAC ACA CAC ACA CAA	44.44	52.2	47.2
U807	AGA GAG AGA GAG AGT	46.67	42.9	37.9
U810	GAG AGA GAG AGA GAG AT	47.06	45.4	40.4
U811	GAG AGA GAG AGA GAG AC	52.94	46.8	41.8

**Table 3:** *E. longifolia* hairy root cultures fresh and dry weights response to a LEDs treatment.

Week	Light emitting diodes treatment	Fresh weight (g)	Dry weight (g)
8	White	2.529 ± 0.36^a^	0.286 ± 0.0 ^a^
	Red	1.745 ± 0.29^a^	0.194 ± 0.02^ab^
	Blue	1.631 ± 0.31^a^	0.194 ± 0.04^ab^
	Blue plus red (1:1)	1.729 ± 0.10^a^	0.176 ± 0.01^b^
	Mint green	1.680 ± 0.33^a^	0.188 ± 0.04^b^
10	White	2.669 ± 0.26^ab^	0.341 ± 0.03^ab^
	Red	1.927 ± 0.08^b^	0.243 ± 0.01^c^
	Blue	2.393 ± 0.28^ab^	0.253 ± 0.01^c^
	Blue plus red (1:1)	2.668 ± 0.17^ab^	0.261 ± 0.03^bc^
	Mint green	3.206 ±0.40^a^	0.368 ± 0.04^a^
12	White	3.611 ± 0.25^a^	0.314 ± 0.01^a^
	Red	2.778 ± 0.14^a^	0.292 ± 0.02^a^
	Blue	2.639 ± 0.28^a^	0.303 ± 0.04^a^
	Blue plus red (1:1)	2.270 ± 0.38^a^	0.255 ± 0.04^a^
	Mint green	2.916 ± 0.84^a^	0.299 ± 0.07^a^

*Notes*:

* Values are shown in mean ± standard error. Each week was analysed separately. Within columns, mean ± standard error with the same alphabet letters indicates no significant difference between means by Duncan’s Test, at *p* ≤ 0.05.

**Table 4:** DAMD-DNA banding profiles of DNA samples obtained from *in vitro Eurycoma longifolia* hairy root cultures with different LED treatments at 8 weeks.

Treatment	Primers	Total number of bands in control (White)	Total number of bands in treatment	Number of monomorphic bands	Number of polymorphic bands	Length of amplified DNA fragments (bp)	SI index
Red	M13	8	8	8	0	400–2,000	1
	M13A	11	12	12	1	300–2,500	1
	URP32F	6	4	5	2	400–800	1
	6_2H_t	7	7	7	0	250–2,500	1
Blue	M13	8	7	8	1	400–2,000	1
	M13A	11	7	8	4	200–1,400	0.89
	URP32F	6	3	4	3	400–800	0.89
	6_2H_t	7	7	7	0	250–2,500	1
Blue plus red (1:1)	M13	8	8	8	0	400–2,000	1
	M13A	11	5	8	4	400–2,500	1
	URP32F	6	5	5	1	400–1,200	0.91
	6_2H_t	7	7	7	0	250–2,500	1
Mint green	M13	8	7	8	1	400–2,000	1
	M13A	11	8	9	3	400–2,500	0.95
	URP32F	6	5	5	1	400–1,200	0.91
	6_2H_t	7	7	7	0	250–2,500	1

Total bands		128	107	116 (90.6%)	22 (17.2%)		

**Table 5:** DAMD-DNA banding profiles of DNA samples obtained from *in vitro Eurycoma longifolia* hairy root cultures with different LED treatments at 10 weeks.

Treatment	Primers	Total number of bands in control (White)	Total number of bands in treatment	Number of monomorphic bands	Number of polymorphic bands	Length of amplified DNA fragments (bp)	SI index
Red	M13	14	13	13	1	350–2,000	0.96
	M13A	12	13	12	1	200–2,900	0.96
	URP32F	7	9	8	2	400–1,400	1
	6_2H_t	7	8	8	0	250–2,500	1
Blue	M13	14	14	14	0	350–2,000	1
	M13A	12	13	12	1	200–2,900	0.96
	URP32F	7	7	7	0	400–1,300	1
	6_2H_t	7	7	7	0	250–2,500	1
Blue plus red (1:1)	M13	14	14	14	0	350–2,000	1
	M13A	12	14	12	2	200–2,900	0.92
	URP32F	7	8	8	1	400–1,400	1
	6_2H_t	7	7	7	0	250–2,500	1
Mint green	M13	14	13	13	1	350–2,000	0.96
	M13A	12	13	12	1	200–2,900	0.96
	URP32F	7	9	8	1	400–1,400	1
	6_2H_t	7	8	8	1	250–2,500	1

Total bands		160	170	163 (100%)	12 (7.5%)		

**Table 6:** DAMD-DNA banding profiles of DNA samples obtained from *in vitro Eurycoma longifolia* hairy root cultures with different LED treatments at 12 weeks.

Treatment	Primers	Total number of bands in control (White)	Total number of bands in treatment	Number of monomorphic bands	Number of polymorphic bands	Length of amplified DNA fragments (bp)	SI index
Red	M13	13	13	13	0	300–1,800	1
	M13A	11	11	11	0	200–2,900	1
	URP32F	4	7	6	3	200–1,200	1
	6_2H_t	7	7	7	0	250–2,500	1
Blue	M13	13	14	13	1	300–1,800	0.96
	M13A	11	11	11	0	200–2,900	1
	URP32F	4	6	6	2	400–1,200	1
	6_2H_t	7	7	7	0	250–2,500	1
Blue plus red (1:1)	M13	13	13	13	0	400–1,800	1
	M13A	11	10	10	1	200–2,900	0.95
	URP32F	4	6	6	2	400–1,200	1
	6_2H_t	7	7	7	0	250–2,500	1
Mint green	M13	13	11	11	2	280–1,800	0.92
	M13A	11	13	13	2	200–2,900	1
	URP32F	4	4	4	0	400–800	1
	6_2H_t	7	8	7	1	250–2,500	1

Total bands		140	148	145 (100%)	14 (10%)		

**Table 7:** ISSR-DNA banding profiles of DNA samplesobtainedfrom *in vitro Eurycoma longifolia* hairy root cultures with different LED treatments at 8 weeks.

Treatment	Primers	Total number of bands in control (White)	Total number of bands in treatment	Number of monomorphic bands	Number of polymorphic bands	Length of amplified DNA fragments (bp)	SI index
Red	N3	2	2	2	0	400–650	1
	N4	4	4	4	0	300–500	1
	U810	3	3	3	0	550–950	1
	N6	10	10	10	0	300–2,900	1
Blue	N3	2	2	2	0	400–650	1
	N4	4	5	5	1	300–800	1
	U810	3	3	3	0	550–950	1
	N6	10	9	9	1	300–2,900	0.95
Blue plus red (1:1)	N3	2	2	2	0	400–650	1
	N4	4	5	5	1	300–800	1
	U810	3	3	3	0	550–950	1
	N6	10	10	10	0	300–2,900	1
Mint green	N3	2	2	2	0	500–850	1
	N4	4	5	5	1	300–800	1
	U810	3	4	4	1	550–950	1
	N6	10	9	9	1	550–1,800	0.95

Total bands		76	78	78(100%)	6(7.89%)		

**Table 8:** ISSR-DNA banding profiles of DNA samples obtained from *in vitro Eurycoma longifolia* hairy root cultures with different LED treatments at 10 weeks.

Treatment	Primers	Total number of bands in control (White)	Total number of bands in treatment	Number of monomorphic bands	Number of polymorphic bands	Length of amplified DNA fragments (bp)	SI index
Red	N3	2	2	2	0	400–600	1
	N4	13	13	13	0	300–1,600	1
	U810	5	1	1	4	550–600	0.33
	N6	8	9	9	1	300–1,400	1
Blue	N3	2	2	2	0	400–600	1
	N4	13	13	13	0	300–1,600	1
	U810	5	5	5	0	350–650	1
	N6	8	9	9	1	300–1,400	1
Blue plus red (1:1)	N3	2	3	3	1	400–600	1
	N4	13	11	11	2	300–1,400	0.92
	U810	5	1	1	4	550–600	0.33
	N6	8	11	11	3	300–1,800	1
Mint green	N3	2	3	3	1	400–600	1
	N4	13	12	12	1	300–1,600	0.96
	U810	5	4	4	1	350–600	0.89
	N6	8	11	11	3	300–1,800	1

Total bands		112	110	110 (98.2%)	22 (19.6%)		

**Table 9:** ISSR-DNA banding profiles of DNA samples obtained from *in vitro Eurycoma longifolia* hairy root cultures with different LED treatments at 12 weeks.

Treatment	Primers	Total number of bands in control (White)	Total number of bands in treatment	Number of monomorphic bands	Number of polymorphic bands	Length of amplified DNA fragments (bp)	SI index
Red	N3	11	10	10	1	200–1,100	0.95
	N4	5	8	5	3	250–1,100	0.77
	U810	2	2	2	0	500–800	1
	N6	13	11	11	2	200–2,900	0.92
Blue	N3	11	11	11	0	200–1,100	1
	N4	5	5	5	0	300–800	1
	U810	2	2	2	0	500–800	1
	N6	13	13	13	0	200–1,400	1
Blue plus red (1:1)	N3	11	8	8	3	400–1,100	0.84
	N4	5	7	5	2	300–900	0.83
	U810	2	1	2	1	500–800	1
	N6	13	11	11	2	250–2,000	0.92
Mint green	N3	11	8	8	3	400–1,100	0.84
	N4	5	6	5	1	300–900	0.91
	U810	2	2	2	0	500–800	1
	N6	13	13	13	0	250–2,000	1

Total bands		124	118	113 (91.12%)	18 (14.5%)		

**Table 10:** Similarity percentages are based on DAMD-DNA banding profiles of DNA samples obtained from *in vitro Eurycoma longifolia* hairy root cultures (ELHRCs) on week 8, 10 and 12.

Week of treatment	Molecular marker	Total no. of bands in control	No. of monomorphic bands	No. of polymorphic bands	Monomorphism (%)	Polymorphism (%)
8	DAMD	128	108	22	90.6	17.2
	ISSR	76	78	6	100.0	7.89
10	DAMD	160	163	12	100.0	7.5
	ISSR	112	110	2	98.2	19.6
12	DAMD	140	145	14	100.0	10.0
	ISSR	124	112	18	91.1	14.5
